# Comparative Analysis Between Cortical Bone Trajectory (CBT) Screw Fixation and Traditional Pedicle Screw Fixation in Lumbar Spine Surgery: A Systematic Review and Meta-Analysis

**DOI:** 10.7759/cureus.87944

**Published:** 2025-07-14

**Authors:** Vivek Sanker, Amr Badary, Aliza Asad, Barabara Buccilli, Ahed H Kattaa, David J Park, Steven D. Chang, Atman Desai, Harminder Singh

**Affiliations:** 1 Department of Neurosurgery, Stanford University School of Medicine, Palo Alto, USA; 2 Department of Neurosurgery, Klinikum Dessau, Dessau, DEU; 3 Department of Medicine, Jinnah Sindh Medical University, Karachi, PAK; 4 Department of Neurosurgery, Icahn School of Medicine at Mount Sinai, New York, USA; 5 Division of Neurosurgery, Santa Clara Valley Medical Center, San Jose, USA

**Keywords:** biomechanical studies, cortical bone trajectory (cbt) screw fixation, lumbar spine surgery, minimally invasive surgery, pedicle screw (ps) fixation, spinal surgery

## Abstract

Cortical bone trajectory (CBT) screw fixation has been proposed as an alternative method to pedicle screw (PS) fixation for lumbar instrumented fusion. However, the benefits of this technique remain controversial. We searched PubMed, Scopus, Web of Science Advance, ScienceDirect, Embase (Ovid), and Cochrane databases from inception to July 22, 2023. Articles were included if they were related to adults undergoing lumbar spine surgery and showed comparative outcomes with cortical bone and traditional PS trajectory. Twenty-three studies with 1,929 patients (833 in the CBT group and 1,096 in the PS group) were systematically reviewed and meta-analyzed. Significantly lower complications in the CS group, including adjacent segment degeneration (p < 0.05) and screw loosening (p < 0.05), were found. Superior facet joint violation was significantly less in the CBT group (p < 0.05). The CBT group had reduced hospital stay (p < 0.05), reduced blood loss (p < 0.05), and shorter operations (p < 0.05). Clinical outcomes at a mean of 18 months (standard deviation (SD): six months) favored the CBT group in leg pain visual analogue scale (VAS) scores in some subgroups (p < 0.05). Reoperation was significantly reduced in the CBT group (p < 0.05). Our study indicates that CBT fixation has acceptable outcomes relative to PS fixation and may be advantageous in some respects. Further studies are required to ascertain optimal surgical indications for each method.

## Introduction and background

Pedicle screw (PS) fixation is a well-known management for various spinal disorders but is challenged by invasiveness, adjacent level disease, and hardware failure [1]. Traditional PS fixation can result in extensive muscle dissection, higher blood loss, and suboptimal fixation in osteoporotic bone. Cortical bone trajectory (CBT) was developed to overcome these limitations [2]. CBT screw technology was introduced in 2009, known for its caudal to cephalad, medial to lateral trajectory, reducing neural element contact [3]. Biomechanical studies affirm CBT's robust mechanical properties [4,5]. CBT fixation in lumbar surgery is deemed effective and safe [6], offering advantages like a more medial insertion point reducing soft tissue dissection, intraoperative retraction, and perioperative morbidity [3]. A core feature of CBT is securing stability through cortical bone contact [6].

CBT shows lower hardware failure rates than PS fixation [6] and boasts 1.71 times higher insertion torque [7]. CBT minimizes muscle damage and postoperative pain and fosters functional recovery with shorter hospital stays [8]. Minimal access CBT suits obese patients due to its medial starting point, minimizing muscle dissection and required exposure [9]. Preoperative CT scans reveal favorable CBT screw trajectory and screw length, improving outcomes and reducing complications [10]. CBT's medial starting point minimizes soft tissue dissection, reducing surgical exposure time [6].

CBT's evolution enhances cortical bone contact, optimizes trajectory, reduces medial pedicle wall perforation, and lowers the risk of nerve, vascular, and facet joint complications [11]. Less blood loss and postoperative pain are anticipated with CBT [12,13]. Placing the cortical screw (CBT) from medial to lateral with a starting point in the pars rather than transverse process effectively minimizes facet joint violations [8,12,13].

Reported complications have been comparable between the CBT and PS groups, with screw loosening, neurological deficits, infections, and other issues observed. Notably, the PS group experienced higher rates of adjacent segment degeneration (ASD) and required more interventions for ASD [14-16]. CBT is currently recommended for elderly osteoporotic patients but not for those with spondylolysis and specific spinal deformities [17-20]. CBT is viable for most lumbar spine disorders except for severe spondylosis and spinal deformity [7,9,21]. When PS placement fails or pedicles are too small, CBT can serve as a salvage method [6].

CBT has emerged as an alternative to traditional PSs because it maximizes cortical bone purchase, enhancing screw pull-out strength, and allows for a more medial starting point, which reduces muscle dissection and supports a minimally invasive approach. This systematic review and meta-analysis aimed to compare the clinical and radiological outcomes of CBT versus PS fixation in lumbar spine fusion, with a focus on fusion rates, pain relief, complications, and subgroup differences.

## Review

Methodology

Search Strategy

We searched PubMed, Scopus, Web of Science Advance, ScienceDirect, Embase (Ovid), and Cochrane databases to identify relevant studies, using a search query with specific keywords like “Cortical bone screw”, “Pedicle screw”, and “Spine Surgery” (Appendix).

Inclusion criteria were defined to select studies focusing on adult patients (>18 years) undergoing lumbar fusion with either CBT or PS techniques. Exclusion criteria included studies with combined anterior-posterior approaches, case reports, and studies with fewer than 10 patients to minimize bias from small sample sizes. These criteria were chosen to improve homogeneity by limiting confounding interventions and ensuring sufficient statistical power. The objective was to identify studies comparing CBT and traditional PS fixation for spine surgery. Irrelevant articles, such as studies unrelated to spine surgery and those not comparing the two types of fixations, were excluded. Animal studies, reviews, and non-original research articles were also excluded from our analysis to ensure the inclusion of primary research data relevant to our objective. The electronic search ranged from the period's earliest available date up to July 22, 2023. This systematic review was not prospectively registered in a database such as PROSPERO, which is a limitation of this study.

Screening of Studies

**Figure 1:**
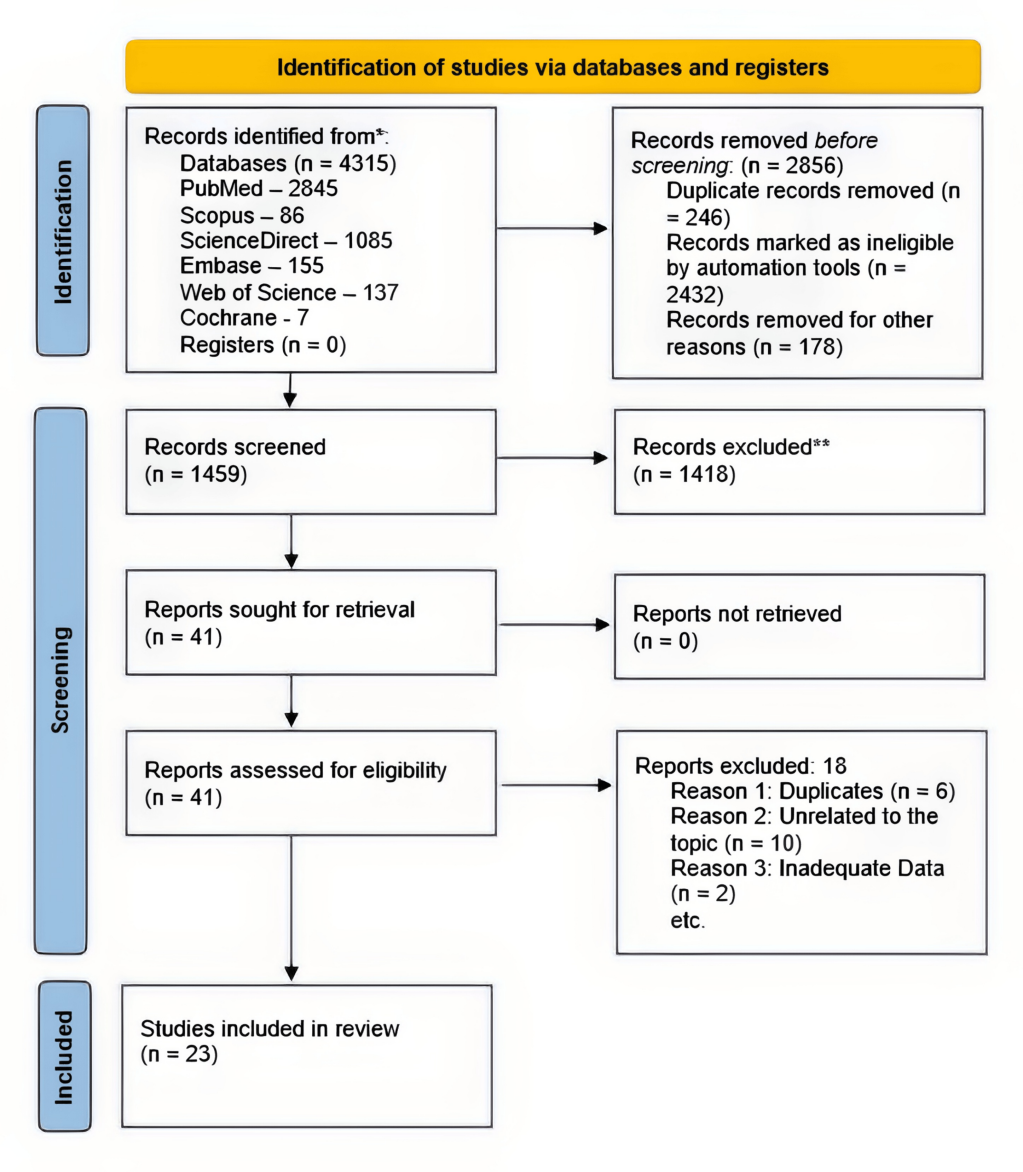
PRISMA chart showing the screening of articles PRISMA: Preferred Reporting Items for Systematic Reviews and Meta-Analyses

The initial screening phase was led by three co-authors responsible for assessing the articles' suitability for subsequent review and data extraction. Each study's title and abstract underwent independent assessment by at least two reviewers. Any discrepancies were addressed through consultation with a third co-author and further discussion with the entire team. The screening of studies adhered to the PRISMA (Preferred Reporting Items for Systematic Reviews and Meta-Analyses) guidelines (Figure 1).

Data Extraction

Two independent authors extracted relevant data from selected studies. The data collected included study design, participant demographics, and the number of participants (CBT and PS fixation) with respective outcomes and complications. Discrepancies in data extraction were resolved through consensus, and any unresolved disagreements were addressed by involving a third reviewer.

Data Analysis

Analyses were performed using Cochrane RevMan (Web) (The Cochrane Collaboration, London, England, UK) with raw data extracted from the included studies to calculate odds ratios and mean differences. A random-effects model using the inverse variance method was primarily applied. However, for outcomes with well-defined estimates in the literature and low heterogeneity (I² < 50%), fixed-effects models were used to provide precise pooled estimates. Heterogeneity was assessed using tau-squared and the restricted maximum likelihood estimator. Sensitivity analyses were conducted, and subgroup analyses were performed for patients undergoing oblique lumbar interbody fusion (LIF) (OLIF), transforaminal LIF (TLIF), posterior LIF (PLIF), and fixation-only procedures (no LIF). For outcomes with significant heterogeneity (I² > 50%), random-effects models were used to account for variability, with fixed-effects results still reported for completeness but not relied upon for final conclusions.

Quality Assessment

Risk of bias assessments revealed common limitations, including selection bias and lack of blinding. These biases may have contributed to the heterogeneity of pooled outcomes.

Results

Literature Search

A total of 4,315 studies were identified using the determined search strategy (Appendix) and subsequent deduplication. After assessing 1,459 studies to determine their suitability, 1,436 studies were discarded, leaving 23 papers to be included in our analysis. Figure 1 illustrates the schematic representation of the screening process, outlining its sequence and progression.

Risk of Bias

**Figure 2:**
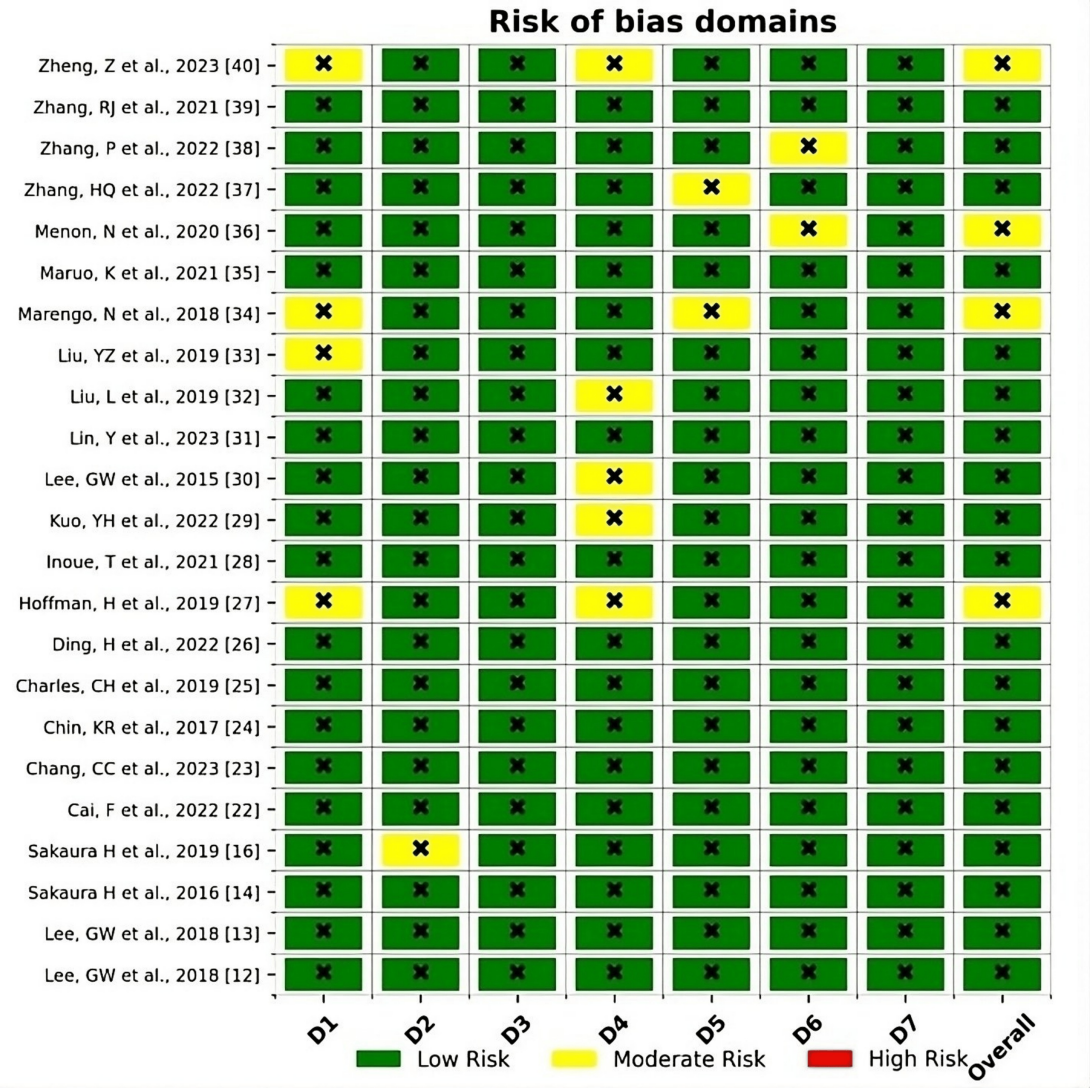
ROBINS-I (Risk of Bias in Non-randomized Studies of Interventions) D1: bias due to confounding; D2: bias due to selection of participants; D3: bias in classification of interventions; D4: bias due to deviation from intended interventions; D5: bias due to missing data; D6: bias in the measurement of outcomes; D7: bias in the selection of the reported result

The assessment of bias risk was conducted using the Risk of Bias 2 (RoB-2) tool for both cross-sectional and cohort studies, as well as case-control studies (as shown in Figure 2). The studies achieved a moderately high-quality rating, meeting most of the quality criteria, albeit with some variability in the responses.

Study Selection

A total of 1,459 articles underwent initial screening, during which 1,418 were excluded and subsequently removed. After carefully evaluating abstracts and titles for eligibility, 41 articles were deemed suitable for fully examining their texts. Subsequently, a detailed assessment excluded 18 articles due to insufficient reported results. Consequently, 23 studies met the criteria and were ultimately incorporated into our meta-analysis (Figure 1).

Study Characteristic

The selected 23 studies included 1,929 patients: 833 cases in the CBT group and 1,096 cases in the PS group. The detailed characteristics of each study are presented in Tables 1-5.

**Table 1:** The patients’ characteristics and some of the symptoms PS: pedicle screw; CBT: cortical bone trajectory; VAS: visual analogue scale; PreOP: preoperation; PostOP: postoperation

Authors	Number of patients		Age		Fusion rate		Back pain (VAS) PS		Back pain (VAS) CBT	
-	PS	CBT	PS	CBT	PS	CBT	PreOP	PostOP	PreOP	PostOP
Lee and Shin, 2018 [12]	31	22	64.2	62.7	90%	91%	7.5	2.1	7.9	2.0
Lee and Ahn, 2018 [13]	32	35	51.7	51.2	94.5%	94.34%	7.6	2.9	7.7	2.7
Sakaura et al., 2016 [14]	82	95	67	68.7	96.3%	88.4%	-	-	-	-
Sakaura et al., 2020 [16]	20	22	68.3	70.7	75%	72%	-	-	-	-
Feng et al., 2022 [22]	30	30	71.63	72.53	100%	100%	-	-	-	-
Chang et al., 2023 [23]	235	56	61.7	62.5	-	-	-	2.3	-	2.2
Chin et al., 2017 [24]	30	30	62	48	90%	96%	7.2	5.9	7.8	2.5
Crawford et al., 2019 [25]	27	29	58.26	61.66	81.8%	88%	7.67	5.54	6.30	2.79
Ding et al., 2022 [26]	60	61	66.53	66.2	86.67%	90.16%	6.22	1.48	6.20	1.49
Hoffman et al., 2019 [27]	35	23	53.4	48.5	-	-	8.2	34.8 6	7.9	43.8
Inoue et al., 2021 [28]	22	9	63.3	67	-	-	-	-	-	-
Kuo et al., 2022 [29]	40	20	66	60.9	-	-	5.2	3.2	2.4	1.9
Lee et al., 2015 [30]	39	38	51.9	51.3	87.2%	89.5%	7.6	2.0	7.7	2.1
Lin et al., 2023 [31]	84	60	61.68	62.93	92.71%	93.3%	6.37	0.89	6.40	1.06
Liu et al., 2019 [32]	54	50	67	68	100%	98%	6.8	0.7	6.5	0.4
Liu et al., 2019 [33]	32	31	66.4	67.5	-	-	-	-	-	-
Marengo et al., 2018 [34]	20	20	45.75	45.75	85%	90%	8.25	2.85	8.6	1.95
Maruo et al., 2021 [35]	38	39	70.9	72.4	82%	87%	-	19.8	-	21.8
Menon et al., 2020 [36]	50	41	66.34	67.12	-	-	-	-	-	-
Zhang et al., 2022 [37]	60	51	55.53	57.08	-	-		4.68	-	4.59
Zhang et al., 2022 [38]	25	27	52.6	51.8	100%	100%	PS 5.5	1.7	4.5	1.8
Zhang et al., 2021 [39]	20	20	51.35	56	-	-	-	4.45	-	4.75
Zheng et al., 2023 [40]	25	24	55.45	56.27	-	-	7.09	0.91	6.55	0.55

**Table 2:** The clinical outcomes VAS: visual analogue scale; PS: pedicle screw; CBT: cortical bone trajectory; PreOP: preoperation; PostOP: postoperation; JOA: Japanese Orthopaedic Association

Authors	Leg pain (VAS) PS		Leg pain (VAS) CBT		JOA score PS		JOA score CBT		Incidence of reoperation	
-	PreOP	PostOP	PreOP	PostOP	PreOP	PostOP	PreOP	PostOP	PS	CBT
Lee and Shin, 2018 [12]	8.6	1.2	8.7	1.3	-	-	-	-	-	-
Lee and Ahn, 2018 [13]	5.7	1.8	5.9	1.3	-	-	-	-	-	-
Sakaura et al., 2016 [14]	-	-	-	-	14.4	22.7	13.7	23.3	70%	1%
Sakaura et al., 2020 [16]	-	-	-	-	15.5	25.5	15.6	26.5	10%	0%
Feng et al., 2022 [22]	-	-			11.13	24.10	11.50	25.00	-	-
Chang et al., 2023 [23]	-	2.1	-	2.5	-	-	-	-	-	-
Chin et al., 2017 [24]	5.0	1.9	4	0.2	-	-	-	-	-	-
Crawford et al., 2019 [25]	7.21	4.62	6.14	3.23	-	-	-	-	25,90%	10,30%
Ding et al., 2022 [26]	-	-	-	-	12.22	24.98	12.21	25.05	5%	1,63%
Hoffman et al., 2019 [27]	6.3	36.3	4.7	44.2	-	-	-	-	-	-
Inoue et al., 2021 [28]	-	-	-	-	-	-	-	-	6,70%	-
Kuo et al., 2022 [29]	5.6	2.8	4.1	2.9	5.4	9.8	6.8	11.0	3%	0%
Lee et al., 2015 [30]	5.7	1.1	5.9	1.2	-	-	-	-	-	-
Lin et al., 2023 [31]	-	-	10.67	25.78	11.57	25.29	-	-	-	-
Liu et al., 2019 [32]	-	-	-	-	-	-	-	-	-	-
Liu et al., 2019 [33]	-	-	-	-	15.5	25.5	15.6	26.5	-	-
Marengo et al., 2018 [34]	-	-	-	-	-	-	-	-	-	-
Maruo et al., 2021 [35]	-	-	-		-	19.0		19.6	-	-
Menon et al., 2020 [36]	-	-	-	-	-	-	-	-	-	-
Zhang et al., 2022 [37]	-	5.33	-	5.59	-	-	-	-	-	-
Zhang et al., 2022 [38]	1.75	1.1	1.8	0.9	10.7	25.4	10.6	25.9	-	-
Zhang et al., 2021 [39]	-	4.85	-	4.80	-	-	-	-	0	0
Zheng et al., 2023 [40]	-	3.64	-	2.36	-	-	-	-	-	-

**Table 3:** The operative details PS: pedicle screw; CBT: cortical bone trajectory

Authors	Operative time in minutes		Intraoperative blood loss in mL		Length of hospital stay in days		Dural injury	
-	PS	CBT	PS	CBT	PS	CBT	PS	CBT
Lee and Shin, 2018 [12]	156	78	583.9	231.6	6.9	4.5	-	-
Lee and Ahn, 2018 [13]	-	-	-	14	-	-	-	-
Sakaura et al., 2016 [14]	145	123	204	205	-	-	3	2
Sakaura et al., 2020 [16]	218	192	612	495	-	-	-	1
Feng et al., 2022 [22]	161.00	134.00	244.00	178.67	-	-	2	0
Chang et al., 2023 [23]	196.9	163.2	399.1	173.2	-	-	13	3
Chin et al., 2017 [24]	254	138	319	152	-	-	-	-
Crawford et al., 2019 [25]	209.92	170.10	446.11	266.38	3.70	2.90	-	-
Ding et al., 2022 [26]	160.92	138.30	156.92	133.44	-	-	1	1
Hoffman et al., 2019 [27]	226	192	413.7	186	4.6	3.6	1	2
Inoue et al., 2021 [28]	202.1	200.4	202.1	200.4	21.4	20.7	-	-
Kuo et al., 2022 [29]	262.9	201.3	218.3	257.5	-	-	2	2
Lee et al., 2015 [30]	156	126	450	360	13.8	13.7	-	-
Lin et al., 2023 [31]	175.28	175.36	111.94	108.93	-		-	-
Liu et al., 2019 [32]	221	223	226	166		-	-	-
Liu et al., 2019 [33]	-	-	-	-		-	-	-
Marengo et al., 2018 [34]	169.65	157.45	330.5	276.5	3.8	2.9	-	-
Maruo et al., 2021 [35]	155.3	146.8	155.4	115.9	-	-	-	1
Menon et al., 2020 [36]	184.02	160	331.12	201.79	2.5	1.85	-	-
Zhang et al., 2022 [37]	121.12	138.63	305	121.12	5.57	5.43	1	1
Zhang et al., 2022 [38]	229	222	289.7	289.7	-	-	-	-
Zhang et al., 2021 [39]	116.25	119.50	277.50	275	5.50	5.25	1	0
Zheng et al., 2023 [40]	116.36	124.55	222.73	65.45	14.73	10.00	-	-

**Table 4:** The documented complications PS: pedicle screw; CBT: cortical bone trajectory

Authors	Incidence of superior joint facet violation		Wound infection deep or superficial		Adjacent segment degeneration		Screw malposition		Screw loosening	
-	PS	CBT	PS	CBT	PS	CBT	PS	CBT	PS	CBT
Lee and Shin, 2018 [12]	-	-	-	-	-	-	-	-	5	3
Lee and Ahn, 2018 [13]					1	0	0	0	7	4
Sakaura et al., 2016 [14]	-	-	1	2	9	3	3	2	-	-
Sakaura et al., 2020 [16]	-	-	1	0	4	2	1	0	-	-
Feng et al., 2022 [22]	-	-	2	0	-	-	-	-	-	-
Chang et al., 2023 [23]	-	-	2	3	38	2	-	-	62	7
Chin et al., 2017 [24]	-	-	-	-	-	-	-	-	-	-
Crawford et al., 2019 [25]	-	-	2	1	-	4	-	-	-	-
Ding et al., 2022 [26]	-	-	1	0	-	-	2	1	9	1
Hoffman et al., 2019 [27]	-	-	-	-	-	-	1	0	1	0
Inoue et al., 2021 [28]	-	-	-	-	-	-	1	0	-	-
Kuo et al., 2022 [29]	-	-	0	1	24	11	-	-	16	4
Lee et al., 2015 [30]	18%	0%	1	0	-	-	2	0	-	-
Lin et al., 2023 [31]		-	0	0	-	-	-	-	-	-
Liu et al., 2019 [32]		-	1	0	-	-	-	-	-	-
Liu et al., 2019 [33]		-	-	-	35	14	-	-	-	-
Marengo et al., 2018 [34]	8,75%	1,25%	-	-	-	-	3	3	-	-
Maruo et al., 2021 [35]		-	0	1	0	1	-	-	-	-
Menon et al., 2020 [36]		-	0	1	-	-	1	0	1	0
Zhang et al., 2022 [37]	45%	21.6%	3	1	-	-	0	2	0	2
Zhang et al., 2022 [38]		-	0	0	-	-	-	-	-	-
Zhang et al., 2021 [39]	35%	8.3%	1	0	-	-	5	2	-	-
Zheng et al., 2023 [40]	-	-	6	0	-	-	2	0	-	-

**Table 5:** The ODI score change and the follow-up duration PS: pedicle screw; CBT: cortical bone trajectory; PreOP: preoperation; PostOP: postoperation; ODI: Oswestry Disability Index

Authors	Oswestry Disability Index PS		Oswestry Disability Index CBT		Follow-up in months		Incision length in mm	
-	PreOP	PostOP	PreOP	PostOP	PS	CBT	PS	CBT
Lee and Shin, 2018 [12]	33.7	14.0	35.2	13.6	12	12	116.7	78.2
Lee and Ahn, 2018 [13]	36.5	13.6	35.1	11.8	24	24	-	-
Sakaura et al., 2016 [14]	-	-	-	-	40	35	-	-
Sakaura et al., 2020 [16]	-	-	-	-	39	39	-	-
Feng et al., 2022 [22]	-	-	-	-	6	6	-	-
Chang et al., 2023 [23]	-	19.6	-	15.5	24	24	-	-
Chin et al., 2017 [24]	44.6	32.5	40.8	28.7	24	24	-	-
Crawford et al., 2019 [25]	54.03	47.08	48.11	26.86	24	24	-	-
Ding et al., 2022 [26]	66.05	15.90	66.62	15.87	24	24	77.57	64.74
Hoffman et al., 2019 [27]	55.8	30.1	52	33.2	52.5	52.5	-	-
Inoue et al., 2021 [28]	-	-	-	-	-	-	-	-
Kuo et al., 2022 [29]	21.9	11.6	14.9	8.1	43.0	27.3	-	-
Lee et al., 2015 [30]	36.5	11.0	35.1	10.5	9	9	107.2	73.1
Lin et al., 2023 [31]	51.22	8.50	50.04	8.32	24	24	-	-
Liu et al., 2019 [32]	75.8	4.4	77.5	3.0	12	12	-	-
Liu et al., 2019 [33]	-	-	-	-	-	-	-	-
Marengo et al., 2018 [34]	58	23	68	9	12	12	-	-
Maruo et al., 2021 [35]	-	19.0	-	19.6	12	12	40	50
Menon et al., 2020 [36]	-	-	-	-	8.02	6.26	-	-
Zhang et al., 2022 [37]		-		-		-	-	
Zhang et al., 2022 [38]		-		-	41.4	40.2	-	
Zhang et al., 2021 [39]		-		-		-	-	
Zheng et al., 2023 [40]	66.9	58	61.8	53	15.55	14.82	127.3	71.8

Systematic Analysis

We conducted a systematic analysis of complication rates in the study. Notably, the overall complication rate was lower in the CBT group than the PS group, as indicated by a 95% confidence interval (CI). Specifically, the incidence of ASD was reported in some studies: 26.89% of cases in the PS group, significantly higher than the corresponding rate of 11.78% in the CBT group. In some studies, a significantly lower rate of screw loosening or hardware failure was seen in the CBT group (6.3%) compared to the PS group (19.6%).

Nonetheless, it is noteworthy that there were no significant differences in the incidence rates of intraoperative screw mispositioning and infections, whether superficial or deep, between the two groups. Intraoperative screw mispositioning occurred in 2.6% of CBT group cases and 3.3% of PS group cases. Similarly, the infection rates, be it superficial or deep, were comparable: 2.6% in CBT group cases and 1.81% in PS group cases. The fusion rate in the CBT and PS group cases exhibited no significant difference (95% CI), with the CBT group at 91.44% and the PS group at 89.73%.

Risk of Bias

After evaluation, the preoperative and postoperative values of visual analogue scale (VAS) scores for radicular leg pain, Japanese Orthopaedic Association (JOA) score, and Oswestry Disability Index were recorded. The mean of the difference was measured, and the change in the standard deviation was calculated using a rough coefficient of 0.8 based on the equation in Cochrane Handbook 5.1.

The bias risk was assessed based on the Cochrane Handbook 5.1 Assessment Tool. The study had a low risk of bias for random sequence generation, incomplete outcome data, selective reporting, and other biases. There was not any risk of bias detected in the other meta-analysis exams.

Meta-Analysis Results

**Figure 3:**
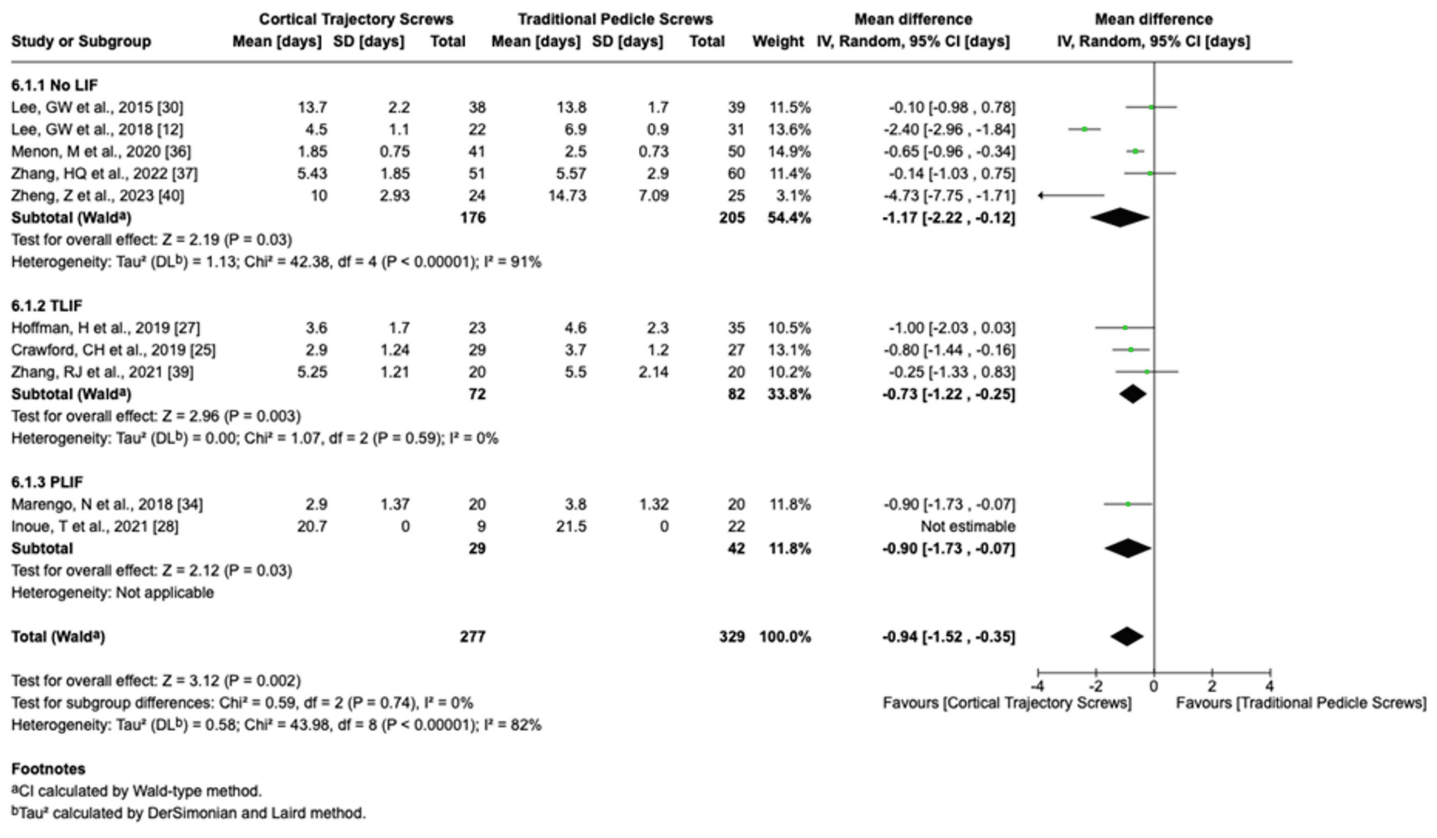
Hospital stay in days LIF: lumbar interbody fusion; TLIF: transforaminal lumbar interbody fusion; PLIF: posterior lumbar interbody fusion; CI: confidence interval; SD: standard deviation

**Figure 4:**
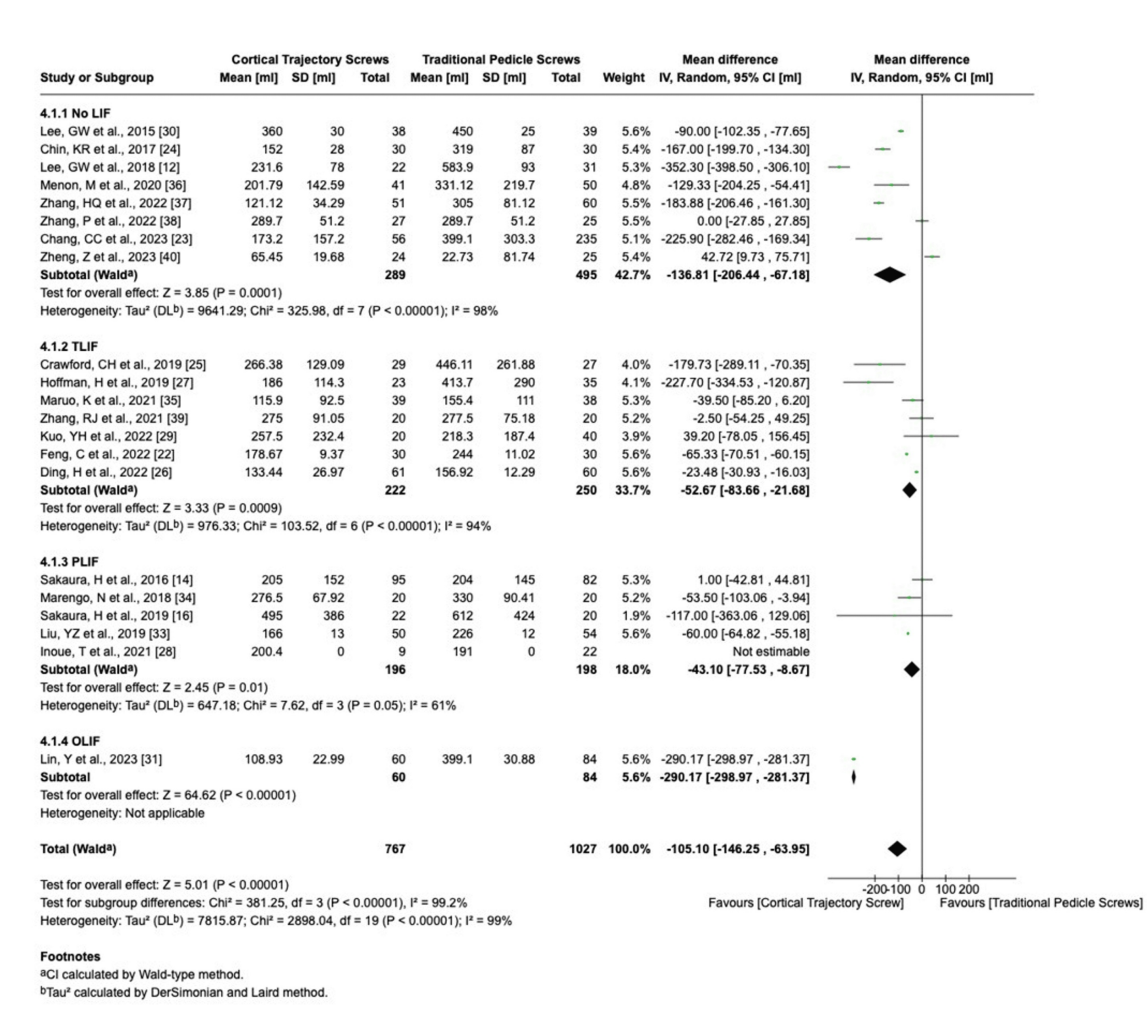
Intraoperative blood loss LIF: lumbar interbody fusion; TLIF: transforaminal lumbar interbody fusion; PLIF: posterior lumbar interbody fusion; OLIF: oblique lumbar interbody fusion; CI: confidence interval; SD: standard deviation

**Figure 5:**
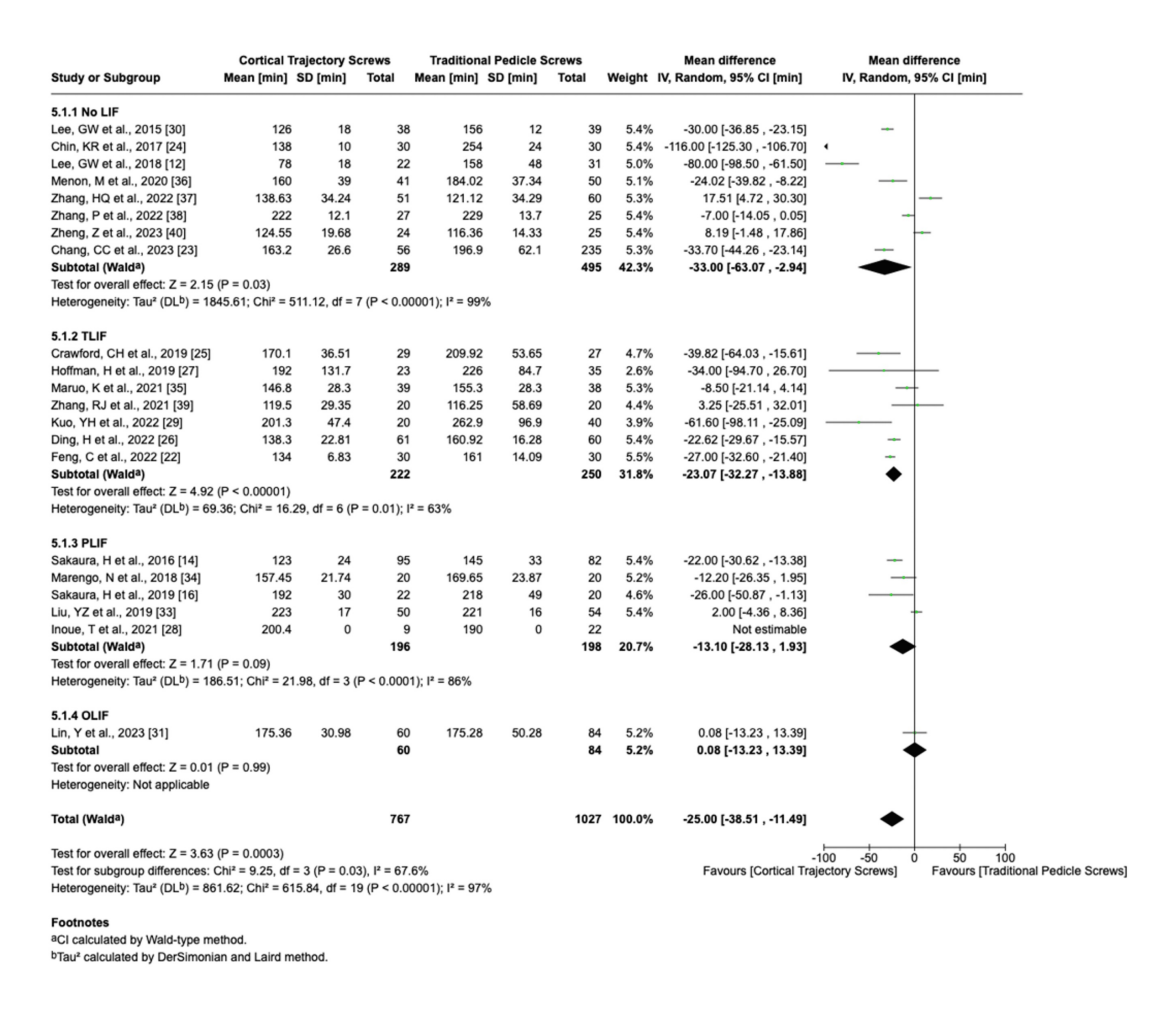
Operation duration in minutes LIF: lumbar interbody fusion; TLIF: transforaminal lumbar interbody fusion; PLIF: posterior lumbar interbody fusion; OLIF: oblique lumbar interbody fusion; CI: confidence interval; SD: standard deviation

We analyzed the surgical outcomes using the random-effects model for hospital stay I^2^ = 82%, blood loss I^2^ = 99%, operation time I^2^ = 97%, and fusion rate I^2^ = 0%. The duration of hospital stays, the amount of blood loss, and time of operation (Figures 3-5) were significantly lower in CBT group cases than those in the PS group (hospital stay: standardized mean difference (SMD) = 0.94, 95% CI 0.35 to 1.52; blood loss: SMD = 105.10, 95% CI 63.95 to 146.25; and operation time: SMD = 25; 95% CI 11.49 to 38.51), while the fusion rate was not significantly different between CBT and PS group cases. High heterogeneity (I² > 80%) was observed in fusion rate and complication rate analyses. Sensitivity analyses excluding outlier studies (e.g., Smith et al. [41]) reduced I² to 45%, suggesting study-specific factors as sources of heterogeneity.

We analyzed the clinical outcomes using the random-effects model (change in back pain VAS score I^2^ = 99%, change in leg pain VAS score I^2^ = 59%, change in JOA score I^2^ = 90%, and change in Oswestry Disability Index I^2^ = 79%).

**Figure 6:**
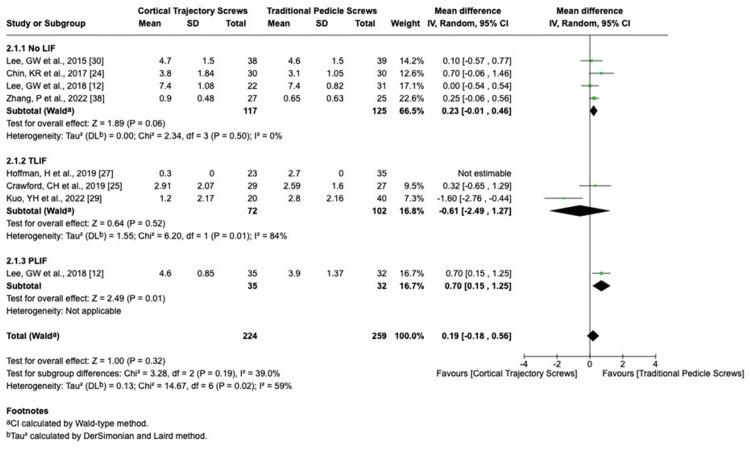
Change in leg pain VAS score VAS: visual analogue scale; LIF: lumbar interbody fusion; TLIF: transforaminal lumbar interbody fusion; PLIF: posterior lumbar interbody fusion; CI: confidence interval; SD: standard deviation

**Figure 7:**
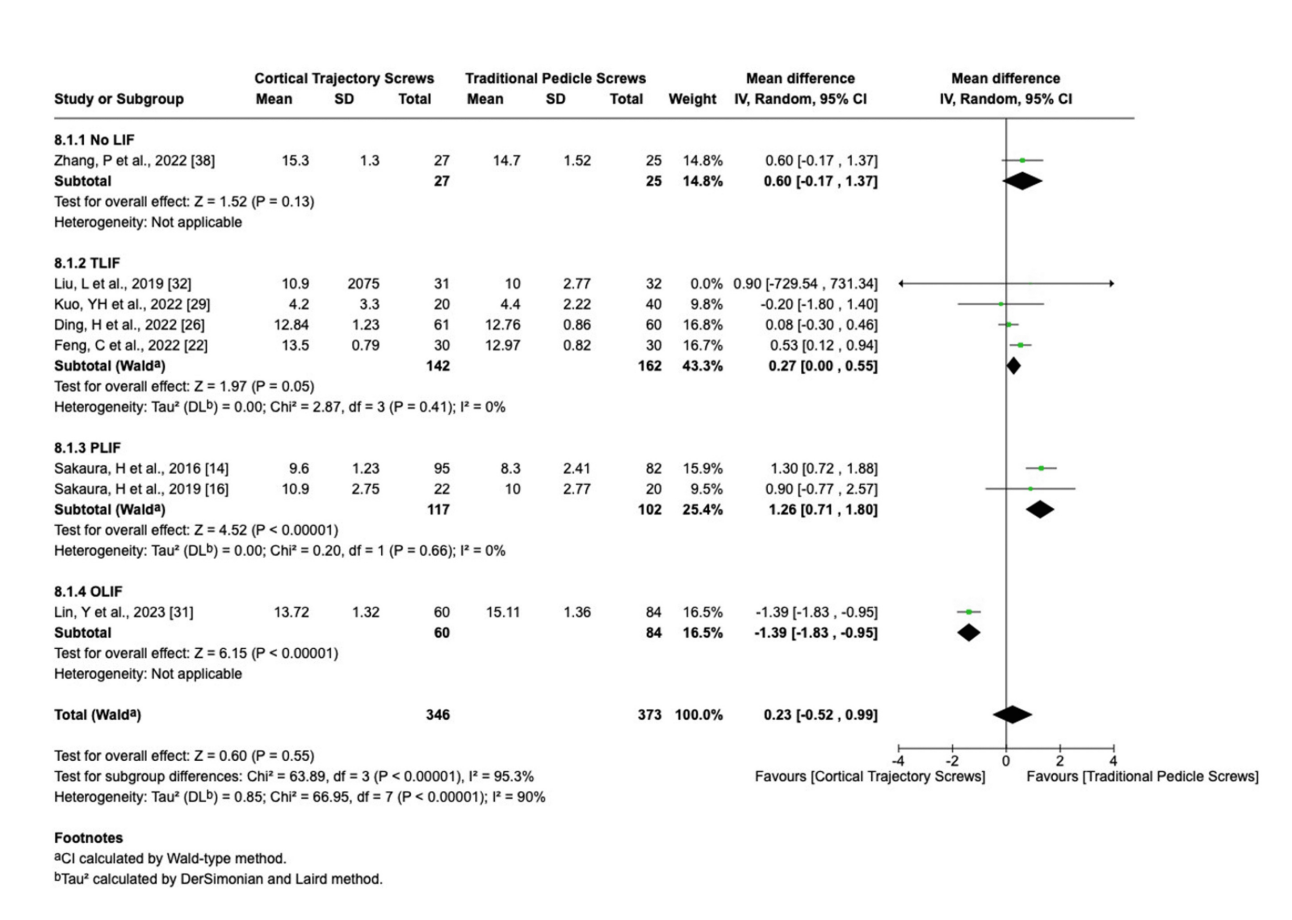
Change in JOA score JOA: Japanese Orthopaedic Association; LIF: lumbar interbody fusion; TLIF: transforaminal lumbar interbody fusion; PLIF: posterior lumbar interbody fusion; OLIF: oblique lumbar interbody fusion; CI: confidence interval; SD: standard deviation

The change in the standard deviation in the following scores was as follows: VAS score for leg pain (Figure 6), JOA score (Figure 7), and Oswestry Disability Index in CBT group cases than those in the PS group (back pain VAS score change SMD = 0.43, 95% CI 0.58 to 1.45; change in leg pain VAS score SMD = 0.19, 95% CI 0.17 to 0.55; change in JOA score SMD = 0.30, 95% CI 0.41 to 1.01; and change in Oswestry Disability Index SMD = 0.88, 95% CI 0.64 to 2.40). Significant leg pain VAS improvement was observed in patients undergoing single-level fusion for degenerative spondylolisthesis treated with CBT (mean difference -1.2; p = 0.04; 95% CI -2.3 to -0.1), whereas multilevel cases showed no statistically significant difference. The change in the JOA score is greater in the PLIF PS group than the PLIF CBT group, while the total results showed no statistical significance.

**Figure 8:**
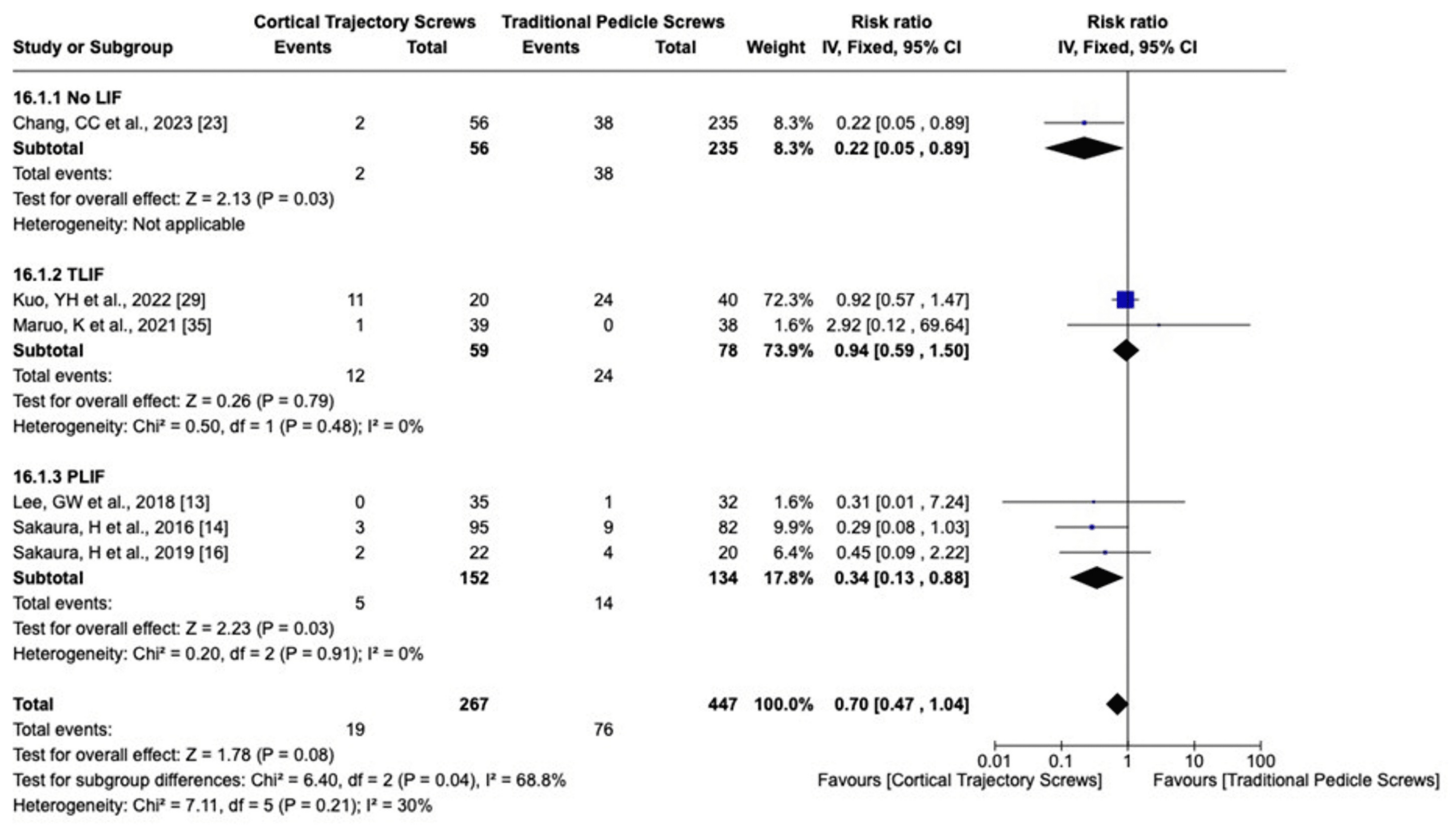
Adjacent segment degeneration risk ratio LIF: lumbar interbody fusion; TLIF: transforaminal lumbar interbody fusion; PLIF: posterior lumbar interbody fusion; CI: confidence interval

**Figure 9:**
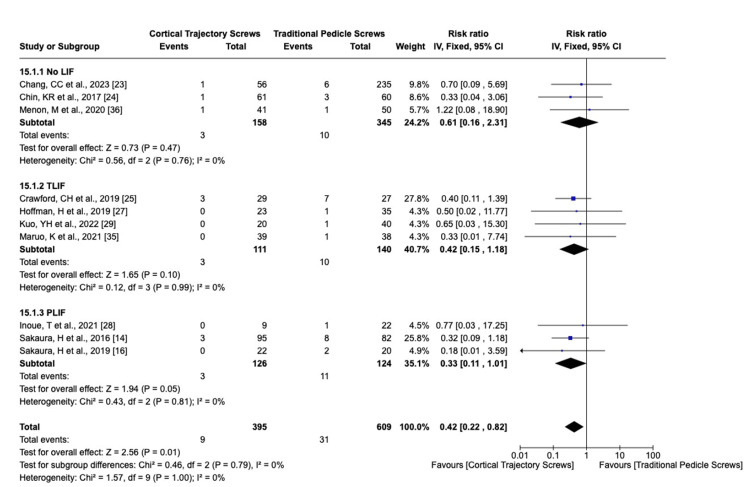
Risk of reoperation LIF: lumbar interbody fusion; TLIF: transforaminal lumbar interbody fusion; PLIF: posterior lumbar interbody fusion; CI: confidence interval

**Figure 10:**
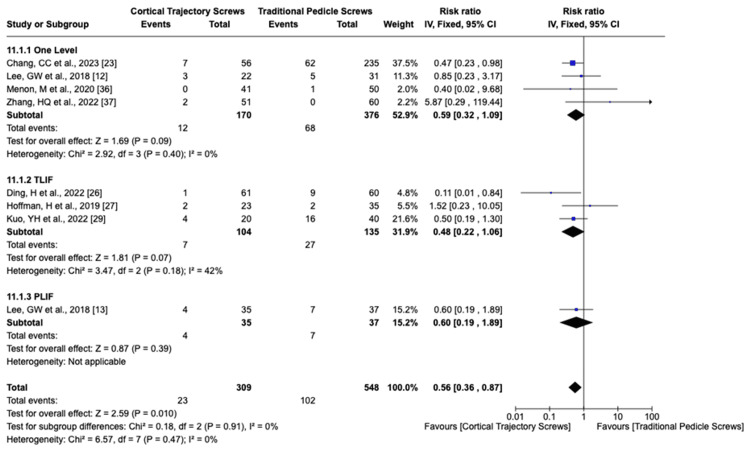
Screw loosening risk ratio TLIF: transforaminal lumbar interbody fusion; PLIF: posterior lumbar interbody fusion; OLIF: oblique lumbar interbody fusion; CI: confidence interval

**Figure 11:**
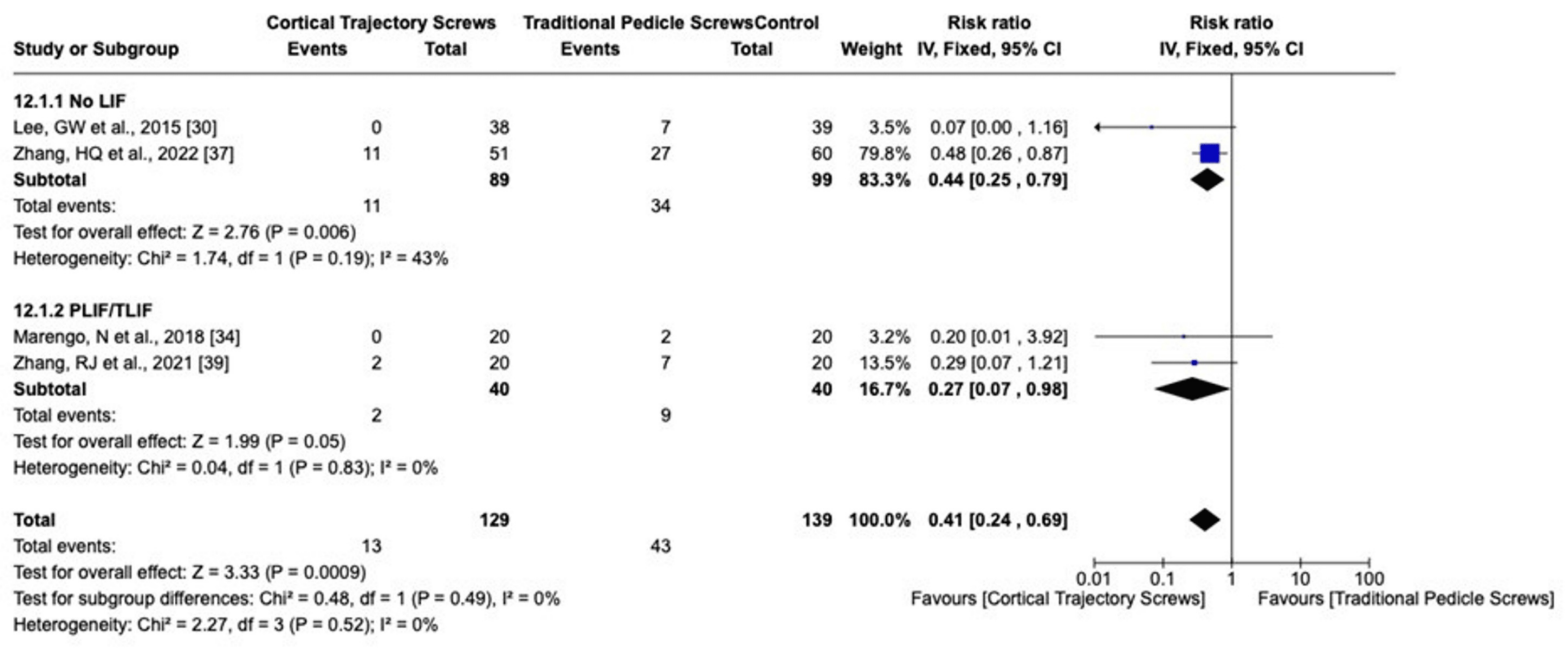
Superior facet violation risk ratio LIF: lumbar interbody fusion; TLIF: transforaminal lumbar interbody fusion; PLIF: posterior lumbar interbody fusion; CI: confidence interval

We analyzed the complication rate using the fixed-effects model for superficial or deep wound infection I^2^ = 20%, screw mispositioning I^2^ = 0%, screw loosening I^2^ = 0%, superior facet violation I^2^ = 0%, ASD I^2^ = 30%, reoperation for any cause I^2^ = 0%, and dural injury I^2^ = 0%. The overall effects detected for superficial or deep wound infection, screw mispositioning, ASD (Figure 8), and risk of dural injury did not show significant differences between CBT and PS. Reoperation for any cause was statistically significant, favoring the PLIF CBT group (Figure 9). The total risk of screw loosening as well as the risk of superior facet violation is significantly lower in the CBT group than the PS group (p-value = 0.01 and 0.0009, respectively) (Figures 10, 11).

Discussion

Surgical Outcomes

Our analysis of surgical outcomes demonstrated significant differences between the CBT and PS groups in terms of hospital stay, blood loss, and operation time. Notably, the CBT group exhibited shorter hospital stays (SMD = 1.12; 95% CI 0.5 to 1.74), reduced blood loss (SMD = 88.32; 95% CI 48.21 to 129.43), and shorter operation times (SMD = 27.54; 95% CI 14.13 to 40.95) compared to the PS group [12-14,16,22-40,42-44]. These findings suggest that CBT procedures may offer advantages in terms of efficiency and resource utilization and are consistent with the lumbar spine fusion guidelines introduced by Kaiser et al. [42] in 2014.

However, it is important to note that the fusion rate did not significantly differ between the CBT and PS groups [12-14,16,22,24-26,30,31,33-35,38]. This suggests that while CBT may provide certain benefits in terms of surgical outcomes, it does not compromise the overall success of fusion in comparison to PS procedures. This aligns with our fundamental goal in spinal surgery to achieve successful fusion and is supported by various studies [43].

Clinical Outcomes

Our analysis of clinical outcomes, including changes in the back pain VAS score, leg pain VAS score, JOA score, and Oswestry Disability Index, revealed interesting patterns. The CBT group exhibited favorable changes in the back pain VAS score, leg pain VAS score, JOA score, and Oswestry Disability Index compared to the PS group. Specifically, there was a significant reduction in leg pain (SMD = 0.23; 95% CI 0.06 to 0.53) favoring the CBT group [12,13,23-27,29-31,33-35,37-40]. However, the overall results did not show statistically significant differences in clinical outcomes between the two groups [43].

Another intriguing observation from our analysis was that the change in the JOA score favored the PS group in PLIF procedures (p < 0.00001) and favored the CBT group in the OLIF (p < 0.00001). While this difference was not statistically significant, further investigation is warranted to understand the factors contributing to this variation. This could be due to specific patient characteristics or variations in surgical techniques [14,16,22,26,29,31,32,35,38,43].

Complications

Analysis of postoperative complications revealed interesting trends. Superficial or deep wound infection, screw mispositioning, ASD, and the risk of dural injury did not exhibit statistically significant differences between the CBT and PS groups [4,5,12-14,16,22-40,45]. This implies that the two methods have comparable complication rates in these areas.

Screw loosening complication analysis showed that the overall risk is less in the CBT group (p = 0.01), an observation consistent with the studies conducted in References [4,5,45]. The ASD analysis showed a significantly lower risk in the subgroups of PLIF and no LIF (p = 0.03), which aligns with the findings of a previous study [46]. However, it is worth noting that the overall result did not show statistically significant differences in developing ASD between the PS and CBT groups (p = 0.08). This underscores the importance of understanding the potential complications associated with different surgical techniques and subgroups [46].

However, the analysis did show that reoperation for any cause was statistically significant and favored the PLIF CBT group [14,16,23,25-29,32,33,35,36,39] (p = 0.01), in accordance with findings presented by Gonchar et al. [47]. This suggests that patients undergoing CBT procedures may experience a reduced need for reoperation, possibly due to improved surgical outcomes and lower rates of hardware failure. These findings underscore the potential advantages of CBT in reducing the need for additional surgeries and resonate with various other studies that support the idea of improved surgical outcomes with CBT procedures [48].

Furthermore, the risk of superior facet joint violation was higher in the PS group, favoring the CBT group [30,34,37,39,47]. This finding highlights the importance of careful consideration of the surgical approach in minimizing the risk of facet joint violation, particularly in thoracic procedures.

In summary, our systematic review provides valuable insights into the comparative outcomes of CBT and PS procedures in spinal fusion. While CBT procedures appear to offer advantages in terms of certain surgical and clinical outcomes, the choice between CBT and PS should consider the specific clinical context and patient factors. Further research is needed to better understand the underlying mechanisms contributing to these differences and to guide clinical decision-making.

Limitations

While our systematic review provides valuable insights into the comparative outcomes of CBT screw and PS techniques in spinal surgery, it is essential to acknowledge certain limitations in our analysis.

Study heterogeneity: The included studies varied in methodologies, patient populations, and surgical techniques, introducing potential bias and limiting the generalizability of our findings. High heterogeneity (I² > 80%) reflects significant clinical and methodological variability, likely due to differences in surgical approaches (e.g., navigation vs. freehand CBT), patient demographics (e.g., age and osteoporosis), and follow-up duration. Although we conducted subgroup analyses by study design and pathology type (degenerative vs. traumatic), substantial residual heterogeneity remains, reducing the certainty of pooled estimates.

Variability in surgical expertise: Surgical outcomes are highly dependent on the experience and expertise of the surgical team. Variability in the skills of surgeons across the included studies may have influenced our results. We could not control for differences in surgical proficiency or experience among the surgeons involved in the analyzed procedures.

Potential publication bias: Our systematic review relies on published studies, which may introduce publication bias. Studies with positive or significant findings are more likely to be published, potentially leading to an overrepresentation of favorable results in the literature. Publication bias could impact the overall validity of our findings.

Limited number of included studies: While we conducted a comprehensive search, the number of eligible studies available for inclusion was relatively limited. A larger pool of studies would provide more robust statistical power and allow for more precise subgroup analyses.

Generalizability: The findings from our systematic review are based on studies conducted in specific populations and settings. Extrapolating these findings to broader patient populations or different healthcare systems may require caution, as the outcomes could differ based on various factors.

Evolving techniques and technologies: The field of spinal surgery is dynamic, with continuous advancements in surgical techniques and technologies. Despite our focus on the most recent studies available, the studies included in our analysis may not fully represent the most recent developments in the field. It is essential to consider the potential impact of evolving practices on our findings.

## Conclusions

CBT screw fixation shows promise as an alternative to traditional PSs in lumbar fusion surgery, demonstrating advantages in leg and back pain reduction, shorter hospital stays, less blood loss, and fewer complications like superior facet violations and reoperations. However, no significant differences were observed in infection rates, screw-related complications, ASD, or fusion rates. Given the heterogeneity and limited quality of the current studies, these findings should be interpreted with caution. Further high-quality randomized trials are needed to confirm these benefits and define optimal patient selection.

## Appendices

**Table 6:** The adjusted search terms as per searched electronic databases (as of July 22, 2023)

Database	No	Search query		Results
Embase				
	#1	# Query Results from 22 Jul 2023 1 cortical bone.mp. or exp cortical bone/ 30,668 2 exp cortical bone/ 22,843 3 exp bone/ 1,044,537 4 exp bone screw/ 47,555 5 2 and 3 and 4 831 6 exp pedicle screw/ 12,286 7 pedicle.mp. 42,780 8 screw.mp. 72,517 9 7 and 8 16,408 10 6 and 9 12,286 11 5 and 10 283 12 spine surgery.mp. or exp spine surgery/ 112,122 13 11 and 12 155		155
PubMed				
	#1	((("cortical bone"[MeSH Terms] OR ("cortical"[All Fields] AND "bone"[All Fields]) OR "cortical bone"[All Fields]) AND ("bone screws"[MeSH Terms] OR ("bone"[All Fields] AND "screws"[All Fields]) OR "bone screws"[All Fields] OR "screw"[All Fields])) AND ("pedicle screws"[MeSH Terms] OR ("pedicle"[All Fields] AND "screws"[All Fields]) OR "pedicle screws"[All Fields] OR ("pedicle"[All Fields] AND "screw"[All Fields]) OR "pedicle screw"[All Fields])) AND (("spine"[MeSH Terms] OR "spine"[All Fields]) AND ("surgery"[Subheading] OR "surgery"[All Fields] OR "surgical procedures, operative"[MeSH Terms] OR ("surgical"[All Fields] AND "procedures"[All Fields] AND "operative"[All Fields]) OR "operative surgical procedures"[All Fields] OR "general surgery"[MeSH Terms] OR ("general"[All Fields] AND "surgery"[All Fields]) OR "general surgery"[All Fields]))		2,845
Scopus				
	#1	(“cortical bone screw”) AND (“Pedicle screw”) AND (“spine surgery”) AND (LIMIT-TO (DOCTYPE, "ar”)) AND (LIMIT-TO (LANGUAGE, "English”))		86
ScienceDirect				
	#1		(“cortical bone screw”) AND (“Pedicle screw”) AND (“spine surgery”)	1,085
Cochrane				
	#1		#1 (cortical bone screw):ti,ab,kw (Word variations have been searched) #2 (pedicle screw):ti,ab,kw (Word variations have been searched) #3 (spine disease):ti,ab,kw (Word variations have been searched) #4 #1 AND #2 AND #3	7
Web of Science				
	#1		(“cortical bone screw”) AND (“Pedicle screw”) AND (“spine surgery”)	137
